# Excess deaths from COVID-19 correlate with age and socio-economic status. A database study in the Stockholm region

**DOI:** 10.1080/03009734.2020.1828513

**Published:** 2020-10-14

**Authors:** Peter Strang, Per Fürst, Torbjörn Schultz

**Affiliations:** a Department of Oncology-Pathology, Karolinska Institutet, Stockholm, Sweden; b R&D Department, Stockholms Sjukhem Foundation, Stockholm, Sweden; c Palliative Care Unit, Stockholms Sjukhem Foundation, Stockholm, Sweden

**Keywords:** COVID-19, excess deaths, hospital care, nursing homes

## Abstract

**Background:**

The COVID-19 pandemic has affected the entire health care system, internationally as well as in Sweden. We aimed to study excess deaths (all death causes, but also COVID-19-related deaths) during the COVID-19 pandemic regarding age, socio-economic status, the situation in nursing homes, and place of death for nursing home residents.

**Design:**

We performed a descriptive regional registry data study using VAL, the Stockholm Regional Council’s central data warehouse, which covers almost all health care use in the county of Stockholm. *T* tests and chi-square tests were used for comparisons.

**Results:**

Compared with 2016–2019, there were excess deaths in March–May 2020 (*p* < 0.0001), mainly explained by COVID-19, but in April there were also unexplained excess deaths. Individuals dying from COVID-19 were older than patients dying from other causes (*p* < 0.0001). There were more patient deaths among people residing in less advantaged socio-economic areas (*p* < 0.0001). Nursing home residents dying from COVID-19 were more often admitted to acute hospitals than residents dying from other causes (*p* < 0.0001). Also, the proportion of admissions of nursing home residents dying from other causes increased from April to May 2020 (*p* < 0.0001).

**Conclusions:**

Dying from COVID-19 mainly affects the elderly, nursing home residents, and persons from less advantaged socio-economic groups. The pandemic has resulted in an increase in acute admissions of dying nursing home residents to acute hospitals.

## Introduction

Although COVID-19 probably already existed during the autumn of 2019, the outbreak in Wuhan, China was formally announced by the WHO in December 2019. Initially, it was uncertain whether the virus would reach countries like Sweden, but in March 2020 it was obvious that it had done so, with the first confirmed death from COVID-19 occurring in the middle of the month.

Soon, it was evident that Sweden had a rapid dissemination of the disease, especially in the Stockholm region that covers about 2.3 million inhabitants. Initially, the major concerns were regarding the capacity and the limits of intensive care units (ICUs) in the region, as well as worries about limited access to personal protective equipment (PPE). However, already from the beginning, it was also clear that frail elderly people with comorbidities had the highest risk of dying from COVID-19 and that age in itself was an established important risk factor (1,2).

In a Swedish context, the impact of socio-economic factors was also apparent in March. Regarding the Stockholm region, patients from less affluent areas seemed to already be overrepresented among deaths at the beginning of the pandemic, and there were alarming early reports stating that individuals from certain non-European backgrounds were overrepresented among the deceased. This was confirmed in a study where deaths in 2020 were compared with deaths in 2016–2019, month by month (3). Immigrants from Somalia, Syria, and Iraq who typically live in less affluent areas had a much higher rate of death than other groups.

For obvious reasons, nursing homes also received a great deal of attention, as a substantial proportion of the deaths occurred in these services among the elderly, frail residents. Due to the organization of Swedish elderly care, with the principle of ‘aging at home’ (*kvarboendeprincipen*), the goal is to stay in one’s own home with the aid of home-help services for as long as possible. A person is accepted for nursing home care after a municipal needs-assessor’s decision, and only when 6–8 scheduled visits per day by home-help services, including nightly visits, are insufficient to support a person with high needs of assistance with activities of daily living (ADL). For this reason, the mean age of the residents is over 85 years. More than 60% suffer from cognitive failure or diagnosed dementia, and most have several comorbidities (4). Moreover, expected survival is limited in nursing homes. In a survey in the Stockholm region, one-tenth of the residents died within a week of admission, one-third within six months, and almost 50% died within the first year (5).

In the county of Stockholm, the municipalities are responsible for nursing homes and staffing, except for physicians who are provided by the Region Stockholm (formerly ‘Stockholm County Council’). Nursing homes provide help with ADL and offer basic health care, when needed. About 36% of all deaths in Sweden take place in nursing homes (6). If the care provided by a nursing home is not sufficient in an individual case, admission to an acute hospital (including a geriatric clinic) is possible. When the pandemic was established, Region Stockholm strengthened the existing services and decided on five levels of care for elderly patients infected with COVID-19: 1) nursing homes for people already residing in such homes; 2) nursing home care with the aid of specialized palliative home care units (with a higher degree of medical staffing); 3) geriatric clinics with designated COVID-19 wards; 4) acute hospitals; and 5) ICUs.

The staffing in nursing homes is basic: a great majority of the staff are assistant nurses, and only about 5% are registered nurses. For this reason, nursing homes provide a basic level of care and nursing, but most cannot offer oxygen therapy or intravenous antibiotics which would require more staffing by registered nurses. If oxygen is needed to support a resident affected by COVID-19 this can, in a limited number of cases, be offered in the nursing home, with or without the help of palliative home care teams. In other cases, acute admission to a geriatric clinic with special COVID-19 wards or to an acute hospital is needed. With this strategy in place and these levels of care, almost 65% of nursing home residents with COVID-19 in the Stockholm region recovered from the disease during the first months, whereas about 35% died from COVID-19, some of whom died after referral to hospitals (7).

The pandemic has led to an urgent need for research data concerning those at risk of dying from COVID-19. However, studying deaths that are attributed to COVID-19 is not simple. First, there was a worldwide delay in testing during February and March 2020, meaning that laboratory-confirmed cases only constituted as little as 10–15% of all cases in some countries (8). Moreover, due to a shortage of tests, only severe hospital cases were initially tested, implying that patients with less obvious symptoms in nursing homes might have been overlooked. A general way to partially overcome this problem is to study excess deaths (all causes of death), which is what has been done to track influenza mortality for more than a century (9,10).

We aimed to study excess deaths (all death causes, but also COVID-19-related deaths) during the COVID-19 pandemic, with special reference to age, socio-economic status, and the situation in nursing homes. A further aim was to study place of death for nursing home residents.

## Patients and methods

The Methods and the Results sections are, when possible, reported based on the Strengthening the Reporting of Observational Studies in Epidemiology (STROBE) criteria (11).

### Study design

The study is based on information retrieved from administrative databases. In Sweden, administrative databases can be used to study health care consumption, place of care, and place of death. This also applies now to COVID-19. In the Stockholm region, all appointments, hospital visits, diagnoses, and major costs are registered and stored in VAL, the Stockholm Regional Council’s central data warehouse. Socio-economic status can also be studied, as Region Stockholm subscribes to Mosaic^TM^, a commercial, internationally used database developed by the company Experian. The Mosaic database can be used for several purposes, but, in the form that it is applied within the Stockholm region, three socio-economic groups are defined, based on neighbourhood characteristics. Thus, the Mosaic groups characterize areas, rather than individuals. These groups or areas are based on several variables, of which education, income, family situation, and living arrangements, and also phase of life, origin, and ethnicity are the most important (12–14).

We conducted a descriptive regional registry data study using VAL, the Stockholm region’s central data warehouse. Within VAL, there are separate registers for outpatient visits to hospitals (OVR) and hospital stays (SLV). In Swedish health care a person’s health care consumption can be followed between different administrative systems as Sweden uses unique personal numbers for each individual. Data in VAL registers are based on these personal numbers but encrypted, meaning that a person’s health care consumption can be followed, without revealing personal identity.

The monthly deaths for January to May 2020 were identified and compared with the corresponding months over four consecutive years, 2016–2019. The data were further analysed in relation to age, sex, living arrangements (residents in nursing homes versus all others), and socio-economic status by means of Mosaic.

### Populations

#### Study population

All monthly deaths registered in VAL databases during January to May 2020 (data retrieved 29 June 2020) were included. In accordance with the guidelines by the Public Health Agency of Sweden (*Folkhälsomyndigheten*), any death with a COVID-19 diagnosis according to ICD-10 should be considered as a death from COVID-19 (15).

#### Reference population

All monthly deaths registered in VAL databases during January to May 2016–2019 (four year-cohorts) were included. Based on these data, mean values with 95% confidence intervals (CI) were calculated. The reason for choosing a mean from the four previous years (instead of just data from the previous year) was to smooth out any short-term spikes, e.g. due to an influenza outbreak.

### Variables

Deaths (all causes) as well as deaths with a COVID-19 diagnosis were used as outcome measures. Age, sex, living arrangements (nursing homes versus all others), and Mosaic groups were used as explanatory variables. The Mosaic methodology is based on the assumption that people tend to settle in neighbourhoods where others are quite similar to themselves, and the final Mosaic groups are based on iterative cluster analyses, based on more than 40 socio-economic variables. Thus, Mosaic provides socio-economic information and allows the council to define and allocate different areas of residence to three different socio-economic classes (Mosaic 1–3), mainly based on income and education, but also, for example, on family situation (single/cohabiting/children, etc.) and living arrangements (owned or rented housing, etc.), phase of life, origin and ethnicity, and degree of urbanization. The county of Stockholm is divided into 1300 small areas, and each area is classified as Mosaic 1, 2, or 3. The three groups are approximately equal in size. Mosaic group 1 refers to persons living in the most affluent areas.

As people residing in nursing homes have moved in from their ordinary homes, this may affect their belonging to a certain Mosaic group, although we know that people prefer a nursing home in their local area. With this in mind, we performed an extra pair-wise comparison of Mosaic groups for individuals during their last year of life, compared to their allocated Mosaic group four years previously when they resided in their ordinary home. The differences on a Mosaic group level differed only with 1–2% (e.g. the proportion of nursing home residents allocated to Mosaic group 2 were 37.3% during their last year of life and 37.1% four years previously). Thus, Mosaic groups were found to be rather stable and reliable enough to be used also when studying nursing home residents.

### Selection bias

#### Drop-outs

As reporting data to VAL constitutes the basis for the respective clinic’s/care unit’s remuneration, data are complete with very few missing values. This means that any individual who has used public health care during the actual year is included in the VAL databases, which is also the case for most forms of private care, as private health care suppliers have economic agreements with the regional council.

#### Immediacy

The data in VAL are updated every month, thus, it is possible to retrieve even very recent data.

#### Nursing home residents

Nursing home residents were identified through registrations of medical interventions by physicians, as such care use is exclusive to nursing home residents and has a unique, identifiable code. It is most unlikely that a nursing home resident does not have a single registration; nursing home residents without registrations were not included in the analysis.

### Study size

The study covers total cohorts, i.e. all deaths (all causes) as well as all reported COVID-19-related deaths during January–May 2020, and data have been compared with data for four similar year cohorts (2016–2019). Therefore, no power calculations were made.

### Statistical methods, missing data

The 95% confidence intervals (95% CI) were calculated. *T* tests and chi-square tests were used to compare proportions. The few missing data were not substituted. The SAS version 9.4 and SPSS version 25 software programs were used for statistics.

### Ethics

The study was approved by the National Ethics Authority (*Etikprövningsmyndigheten*, Dnr 2020–02186).

## Results

### Excess deaths (all causes) January–May 2020 compared with 2016–2019

#### All deaths

The mean age of all the deceased from January to May 2020 was 79.5 years (median 83 years), which was higher than for the deaths during the corresponding months in 2016–2019, 78.8 years (median 82 years), *p* < 0.0001. In 2020, 49.6% were female, compared with 52.1% for 2016–2019 (chi-square = 18, 1 df), *p* < 0.0001.

In a first comparison, death rates for January to May 2020 were compared with corresponding calculated means and 95% CI for the corresponding months in 2016–2019. Whereas January and February were similar to the calculated means, the proportions of deaths were significantly higher for March, April, and May (23%, 113%, and 44%, respectively), *p* < 0.0001, for each comparison (Table 1). Regarding age groups, only patients over 80 years of age had excess deaths in March, whereas all the studied age groups were affected in April. In May, excess deaths were mainly attributed to patients aged 70–79 years and to those aged 80 years or more (Table 1).

**Table 1.** Mortality and age categories.

	March				April				May			
	2016–2019 *n*, mean (95% CI)	2020	Excess mortality	Significance^a^ (chi-square)^b^	2016–2019 *n*, mean (95% CI)	2020	Excess mortality	Significance^a^ (chi-square)^b^	2016–2019 *n*, mean (95% CI)	2020	Excess mortality	Significance^a^ (chi-square) ^b^
Monthly mortality	1474 (1326–1622)	1819	23%	*** (38.9)	1376 (1300–1452)	2934	113%	***(1020.5)	1287 (1244–1330)	1860	44%	*** (147.4)
Age categories												
60–69 years	154 (134–174)	153	−1%		145 (126–165)	246	69%	**(44.8)	151 (125–177)	162	7%	
70–79 years	347 (302–391)	378	9%		323 (261–384)	632	96%	***(156.7)	306 (279–333)	406	33%	*** (13.7)
≥80 years	840 (768–911)	1141	36%	*** (46.6)	779 (712–846)	1886	142%	***(821.1)	694 (645–743)	1156	67%	*** (168.1)

The proportions of deaths were significantly higher for march, april, and may (23%, 113%, and 44%, respectively). the chi-square values were well below the limit for *p* < 0.001 (chi-square = 10.83) in all monthly comparisons.

^a^ Levels of significance: *p < 0.05; **p < 0.01; ***p < 0.001.

^b^ In all chi-square comparisons: 1 df (degree of freedom), meaning that the chi-square value for *p* < 0.05 is 3.84; *p* < 0.01 is 6.64; and for *p* < 0.001 is 10.83.

#### Nursing homes versus other places of death

The proportion of patients dying in nursing homes as a fraction of all deaths in 2020 was 32% in March, 43% in April, and 34% in May. When specifically studying the percentage of excess deaths in nursing homes, compared with deaths in 2016–2019, the proportions were found to be significantly higher: 11% in March, 167% in April, and 46% in May (Table 2). There were also excess deaths (all causes) for March–May, for those dying in other places than nursing homes. In March, the excess deaths (%) were higher for other places of deaths than nursing homes, 30% versus 11%, whereas the situation was reversed for April. For details, see Table 2.

**Table 2.** Proportion of all deaths (all causes), and excess deaths (all causes) in March–May 2020, in comparison with data for respective month in 2016–2019, in nursing homes versus in all other places of death.

	Nursing homes			All others		
Month	2016–2019 (95% CI)	2020	Excess mortality	2016–2019 (95% CI)	2020	Excess mortality
March	531 (482–580)	590	11%^a^	944 (828–1059)	1229	30%^a^
April	475 (437–511)	1269	167%^a^	898 (852–951)	1665	85%^a^
May	428 (368–488)	625	46%^a^	855 (795–924)	1235	44%^a^

^a^ *p* < 0.05 in all comparisons between 2016–2019 and 2020.

### Dying from COVID-19

The mean age of patients who died with a COVID-19 diagnosis was 81 years (median 83 years), which was higher than for other causes (Table 3); 45% were female, compared with 51% in 2016–2019 (chi-square = 23, 1 df), *p* < 0.0001.

**Table 3.** Age at death.

	Cause of death		*p* Value
	COVID-19, Mean (median)	Other causes, Mean (median)	
All patients (years)	81.0 (83)	79.2 (82)	<0.0001
Female patients (years)	84.2 (86)	82.0 (86)	<0.0001
Male patients (years)	78.3 (80)	76.3 (79)	<0.001

Patients dying from COVID-19 were significantly older than patients dying from other causes.

The proportions of deaths with a registered COVID-19 diagnosis compared with all deaths were 10% for March, 37% for April, and 32% for May.

### COVID-19 and socio-economic status

#### Per month

When stratifying for socio-economic Mosaic groups, where Mosaic group 1 represents individuals from the most affluent and Mosaic 3 the least affluent socio-economic areas, there were significant differences as regards COVID-19-related deaths for each month during March to May 2020, with more deaths in Mosaic group 3 (Table 4).

**Table 4.** COVID-19-related deaths per month during march–may 2020, in relation to socio-economic mosaic groups 1 and 3.

Month	Mosaic group 1	Mosaic group 3	Chi-square (1 df)	*p* Value
March	36/703478	91/715494	22.9	<0.0001
April	230/703576	451/715666	68.0	<0.0001
May	163/703508	226/715875	9.2	0.003

There were more COVID-19-related deaths in mosaic group 3.

#### Per age group

COVID-19-related deaths were also calculated in relation to every 1000 inhabitants, stratified both for Mosaic groups and for age groups. Data for the most affected month, April 2020, are presented in Table 5. Deaths were consistently higher in Mosaic group 3 compared with Mosaic group 1.

**Table 5.** COVID-19-related deaths/1000 inhabitants in april 2020.

Age group (years)	Mosaic group	Deaths *n*	Deaths/1000 inhabitants	Chi-square (Mosaic 1 versus 3)	*p* Value (Mosaic 1 versus 3)
40–59	1	11	0.05		
	2	14	0.06		
	3	26	0.15	8.6	0.003
60–69	1	17	0.25		
	2	28	0.31		
	3	54	0.86	22.4	<0.00001
70–79	1	58	1		
	2	95	1.19		
	3	110	2.12	22.5	<0.00001
≥80	1	143	5		
	2	274	6.98		
	3	265	7.88	19.8	<0.00001

For each age group, the proportion of deaths was significantly higher in mosaic group 3, compared to mosaic group 1.

### Excess deaths not explained by COVID-19

April was the most affected month, with 1096 confirmed COVID-19 deaths and a total of 2934 deaths. When removing the number of those who died from COVID-19, the remaining number is 1838 deaths in April, which is significantly higher than for the reference years (95% CI 1300–1452).

### Nursing home residents: changes in place of care during the last two weeks of life

Changes in place of care for nursing home residents were mapped for the last two weeks of life, for the months March–May. In a first comparison, we studied trends for 2016–2019 compared with 2020. For the period 2016–2019, 28.3% (95% CI 26.7%–30.0%) had at least one change as regards place of care, compared with 15.2% for the corresponding period in 2020 (chi-square = 162, 1 df; *p* < 0.0001) (Table 6).

**Table 6.** Proportion of nursing home residents with at least one change of place of care during their last 2 weeks of life.

2016–2019, March–May, % (95% CI) with changes in place of care	2020, March–May, % with changes in place of care	Chi-square (1 df)	*p* Value
28.3% (26.7%–30.0%)	15.2%	162.0	<0.0001

March–May 2016–2019 is compared with March–May 2020.

In a second comparison, we did a more detailed comparison for 2020, where we compared those who had died with a COVID-19 diagnosis with all others. For the whole period (March–May), 24% and 12% of those who died with versus without a COVID-19 diagnosis had at least one change of care (chi-square = 59.8, 1 df; *p* < 0.0001). For details, see Table 7.

**Table 7.** Proportion of nursing home residents with at least one change of place of care during their last 2 weeks of life, March–May 2020.

Month	COVID-19 deaths, *n* (%) with changes in place of care	Other causes, *n* (%) with changes in place of care	Chi-square (1 df)	*p* Value
March	22/39 (56%)	111/551 (20%)	27.9	<0.0001
April	99/449 (22%)	43/820 (5%)	80.8	<0.0001
May	48/213 (22%)	55/412 (13%)	8.6	0.003
March–May	169/701 (24%)	209/1783 (12%)	59.8	<0.0001

Residents dying from COVID-19 are compared with residents dying from other causes.

Residents dying from causes other than COVID-19 had more changes of place in May than in April (Table 7). Whereas 5% were referred acutely during the last two weeks of life in April, the corresponding figure had increased to 13% in May (chi-square = 24.6, 1 df; *p* < 0.0001).

### Place of death for nursing home residents

When specifically studying the proportion of nursing home residents who eventually died either in an acute hospital or in a geriatric hospital ward, the proportion for residents dying from COVID-19 during March–May was 19%, and for residents dying from other causes 5% (chi-square = 109, 1 df; *p* < 0.0001) (Table 8).

**Table 8.** Proportion of nursing home residents who eventually died in acute hospitals or geriatric wards, March–May 2020.

Months	COVID-19 deaths, *n* (%) hospital deaths	Other causes, *n* (%) hospital deaths	Chi-square (1 df)	*p* Value
March–May	132/701 (19%)	96/1687 (5%)	109.1	<0.0001

Residents dying from COVID-19 are compared with residents dying from other causes.

## Discussion

Our register data showed significant excess deaths (all causes) for each of the months of March to May, with peak values for April. The excess deaths correlated with more advanced age and consequently also with being resident in a nursing home, as the mean age of Swedish nursing home residents is about 86 years. There was also a correlation with excess COVID-19-related deaths and lower socio-economic status as measured by the Mosaic groups, in good agreement with a detailed report from Region Stockholm (16). An additional finding was that nursing home residents dying from COVID-19 were more often admitted to hospitals than residents dying from other causes.

Our data are in good agreement with similar data from other countries who also report excess deaths, not only related to a COVID-19 diagnosis but also to other causes (9,10,17–19). Such unexplained deaths were also seen in the Stockholm region in April, the most affected month. Excess deaths related to other causes are believed to occur indirectly through delayed care for acute emergencies, exacerbations of chronic diseases, and psychological distress (e.g. drug overdoses) (17).

Age has been a known risk factor already from the start of the epidemic (1,2). For this reason, the Swedish strategy attempted to protect citizens above the age of 70 years from contact with others through a number of recommendations aimed at social distancing. Still, our data show that the COVID-19-related excess mortality in the peak month (April) was most pronounced in these groups, with 86% of the COVID-19-related deaths in persons over 70 years of age, in good agreement with other data from Region Stockholm (16). A disproportionately large proportion of these individuals were residents in nursing homes compared with other living arrangements. However, the proportion dying in nursing homes with a COVID-19 diagnosis (37% in April), is similar to the data from other countries, with figures ranging from 19% in Hungary to 62% in Canada (20). Moreover, people dying from COVID-19 were older than people dying from other causes.

Socio-economic status in itself, as well as belonging to a minority, has been associated with a higher risk of contracting COVID-19 and, also, with a higher risk of death (16,19,21–23). In many studies, socio-economic status and belonging to a minority are strongly intercorrelated, but the risk of contracting COVID-19 and dying from the disease is not explained merely by socio-economic status or comorbidities. As shown by Lassale et al. in their study, black individuals had an increased risk of COVID-19 infection compared with white individuals, even when adjusting for age, sex, and other potential explanatory factors which included neighbourhood deprivation, household crowding, smoking, body size, inflammation, glycated haemoglobin, and mental illness (22). Similar data have been published by Williamson et al. (23).

Sweden has a relatively large proportion of immigrants, with approximately 20% of the population born abroad (24). In a recent study, the number of deaths was 220% higher for immigrants from Somalia, Syria, and Iraq in 2020, compared with deaths in previous years, and much higher than for individuals born in Sweden (3). In our current study, we used Mosaic groups instead of comparing immigrants with persons born in Sweden. Mosaic includes immigration status as one of the defining variables, but also several other unrelated variables, of which education, income, family situation, and living arrangements are the most salient factors. For each age group, COVID-19-related deaths were significantly more frequent for persons living in Mosaic group 3 areas than in Mosaic group 1 areas.

In Sweden, there has been an animated debate concerning the optimal place of care for nursing home residents who are infected with COVID-19, especially considering the high number of deaths in nursing homes. Critics have argued that ‘no one is being admitted to hospitals’, despite inadequate access to oxygen treatment in nursing homes (25). Care of residents with COVID-19 in nursing homes has even been compared with euthanasia by public persons, in an infected debate (26). Therefore, according to the critics, more admissions would mean more saved lives. Others have argued that most of these frail, dying, nursing home residents would not benefit from an acute admission, as an acute transfer from a well-known environment often triggers acute delirium in this patient group and they would not benefit from hospital care.

For this reason, it was of interest to study whether, and to what degree, hospital admissions were made for dying nursing home residents. When comparing the proportion of all changes in place of care in nursing homes in 2020 with figures from previous years (2016–2019), it is obvious that the total number of changes was lower in 2020, implying that there was a general reluctance to admit dying residents to acute hospitals. However, data show that, in 2020, a substantially larger proportion of residents dying from COVID-19, compared with residents dying from other causes, were referred to acute hospitals or geriatric hospital wards. An interesting finding is that although 24% of those dying from COVID-19 and 12% of the others were acutely admitted to hospitals during their last two weeks of life, only 19% and 5% of these patients actually died in hospitals, implying that some of them were sent back to the nursing homes. Therefore, it is not possible to draw any conclusions about the net benefits, i.e. how many lives were saved by acute admissions and how many died in the unfamiliar hospital environment. Estimates by individual providers of nursing home care say that relatively few of those admitted to acute hospitals survived (7).

Because the debate in the media included a great deal of criticism of nursing home care, we also decided to study to what degree nursing home residents dying from *other* causes were acutely admitted to other services during the last two weeks of life, i.e. in the dying phase. We found that whereas only 5% of these residents were admitted to hospitals in April, the figure was significantly higher in May, 13%. A possible explanation is that the general discussion, where the medical competence of nursing homes was questioned in the media, affected the public’s view on nursing home care in general, with subsequent requirements for referrals to hospitals.

Our analyses were based on administrative data from the regional VAL databases. As all health care, with very few exceptions, is financed by taxes and reporting to the VAL databases is a mandatory basis for remuneration, the data have extremely few missing values. This is also the case for private health care, as most of the private care providers have agreements with Region Stockholm.

A possible limitation is that only persons with health care utilization provided by the regional council are registered. This means that basic care in nursing homes, care that is provided by the municipalities, is not registered. However, as all consultations and medical interventions by physicians are provided by the regional council, these interventions are registered with an identifiable coding. In this way, nursing home residents were indirectly identified, but we might have missed some cases.

Another possible limitation is that people in nursing homes that are located in certain socio-economic Mosaic areas (e.g. Mosaic group 2 area) might have moved there from another area belonging to a different Mosaic group. However, specifically for this study, we have compared the actual Mosaic allocation for nursing home residents with their previous Mosaic allocation four years earlier (see Methods section) and found only minor differences, in the range of a few percent. Thus, Mosaic groups are rather stable and reliable enough also when studying nursing home residents.

Finally, we used the definition proposed by the Public Health Agency of Sweden, that any death with an ICD-10 code of COVID-19 should be counted as a death from COVID-19. Future studies will show to what extent people actually died from COVID-19 or died from other causes with COVID-19 as a secondary diagnosis.

## Conclusions

Dying from COVID-19 mainly affects the elderly, and those dying from COVID-19 are, in fact, older than those dying from other causes. Nursing home residents as well as elderly individuals from less advantaged socio-economic groups are at a higher risk.

The pandemic has changed the patterns of care: a higher proportion of nursing home residents with severe forms of COVID-19 are referred to hospitals, and this has, in turn, also affected decisions for residents dying from other causes, with more acutely dying persons admitted to acute hospitals.

## References

[1] Wu C, Chen X, Cai Y, Xia J, Zhou X, Xu S, et al. Risk factors associated with acute respiratory distress syndrome and death in patients with coronavirus disease 2019 pneumonia in Wuhan, China. JAMA Intern Med. 2020;180:934–43. 32167524 10.1001/jamainternmed.2020.0994 PMC7070509

[2] Zhou F, Yu T, Du R, Fan G, Liu Y, Liu Z, et al. Clinical course and risk factors for mortality of adult inpatients with COVID-19 in Wuhan, China: a retrospective cohort study. Lancet 2020;395:1054–62. 32171076 10.1016/S0140-6736(20)30566-3 PMC7270627

[3] Hansson E, Albin M, Rasmussen M, Jakobsson K. [Large differences in excess mortality in March-May 2020 by country of birth in Sweden]. Lakartidningen 2020;117:20113. 32619245

[4] Bjork S, Juthberg C, Lindkvist M, Wimo A, Sandman PO, Winblad B, et al. Exploring the prevalence and variance of cognitive impairment, pain, neuropsychiatric symptoms and ADL dependency among persons living in nursing homes; a cross-sectional study. BMC Geriatr. 2016;16:154. 27549203 10.1186/s12877-016-0328-9 PMC4994232

[5] Schon P, Lagergren M, Kareholt I. Rapid decrease in length of stay in institutional care for older people in Sweden between 2006 and 2012: results from a population-based study. Health Soc Care Community. 2016;24:631–8. 25944315 10.1111/hsc.12237

[6] SRPC Årsrapport för svenska palliativregistret 2018 [Annual report from the Swedish registry of palliative care 2018]. 2018 Available at: http://media.palliativregistret.se/2019/09/%C3%85rsrapport-2018-.pdf (last accessed 22 July 2020).

[7] Amer S, Molnar C, Tuutma M, Metzner C, Tegman P, Taranger M, et al. Almost two-thirds of the elderly with COVID-19 surviving in nursing homes. Lakartidningen 2020;117:20104. 32594470

[8] Li R, Pei S, Chen B, Song Y, Zhang T, Yang W, al. Substantial undocumented infection facilitates the rapid dissemination of novel coronavirus (SARS-CoV-2). Science. 2020;368:489–93. 32179701 10.1126/science.abb3221 PMC7164387

[9] Weinberger DM, Chen J, Cohen T, Crawford FW, Mostashari F, Olson D, et al. Estimation of excess deaths associated with the COVID-19 pandemic in the United States, March to May 2020. JAMA Intern Med. 2020;e203391. 10.1001/jamainternmed.2020.3391 PMC7330834 32609310

[10] Weinberger DM, Cohen T, Crawford FW, Mostashari F, Olson D, Pitzer VE, et al. Estimating the early death toll of COVID-19 in the United States. bioRxiv. 2020.

[11] Vandenbroucke JP, von Elm E, Altman DG, Gøtzsche PC, Mulrow CD, Pocock SJ, STROBE Initiative, et al. Strengthening the Reporting of Observational Studies in Epidemiology (STROBE): explanation and elaboration. Epidemiology. 2007;18:805–35. 18049195 10.1097/EDE.0b013e3181577511

[12] InsightOne. Experia MIS MosaicTM Sweden. 2015.

[13] Dahlén E, Komen J, Jonsson EW, Almqvist C, Kull I, Wettermark B. Eliminated patient fee and changes in dispensing patterns of asthma medication in children – an interrupted time series analysis. Basic Clin Pharmacol Toxicol. 2019;125:360–9. 31188534 10.1111/bcpt.13268

[14] Sharma A, Lewis S, Szatkowski L. Insights into social disparities in smoking prevalence using Mosaic, a novel measure of socioeconomic status: an analysis using a large primary care dataset. BMC Public Health. 2010;10:755. 21138555 10.1186/1471-2458-10-755 PMC3016386

[15] Folkhälsomyndigheten (FHM) [Public Health Agency of Sweden]. Information om datakällor [Information on data sources]. From: Bekräftade fall i Sverige [Confirmed cases in Sweden]. 2020 Available at: https://www.folkhalsomyndigheten.se/smittskydd-beredskap/utbrott/aktuella-utbrott/COVID-19/statistik-och-analyser/bekraftade-fall-i-sverige/

[16] Lager A, Tynelius P, Walander A, Nederby Öhd J, Ponce de Leon A, Zhou M, et al. COVID-19 i Stockholms län till och med mitten av juni 2020. Förloppet och den geodemografiska spridningen [COVID-19 in Stockholm County until. June 2020. Course and geodemographic spread]. Centrum för epidemiologi och samhällsmedicin. Region Stockholm. Rapport 2020:6. 2020 Available at: https://ces.sll.se/globalassets/verksamheter/forskning-och-utveckling/centrum-for-epidemiologi-och-samhallsmedicin/folkhalsoguiden/rapporter-och-faktablad/rapport-2020.6-COVID-19-i-stockholms-lan-till-och-med-mitten-av-juni-2020_uppdaterad-2020-07-13.pdf (last accessed 30 August 2020).

[17] Woolf SH, Chapman DA, Sabo RT, Weinberger DM, Hill L. Excess deaths from COVID-19 and other causes, March-April 2020. JAMA 2020;324:510–3. 32609307 10.1001/jama.2020.11787 PMC7330820

[18] Mannucci E, Nreu B, Monami M. Factors associated with increased all-cause mortality during the COVID-19 pandemic in Italy. Int J Infect Dis. 2020;98:121–4. 32599284 10.1016/j.ijid.2020.06.077 PMC7319652

[19] Friedman J, Calderon-Villarreal A, Bojorquez L, Hernandez CV, Schriger DL, Hirashima ET. Excess out-of-hospital mortality and declining oxygen saturation: the sentinel role of EMS data in the COVID-19 crisis in Tijuana, Mexico. medRxiv. 2020. 10.1016/j.annemergmed.2020.07.035 PMC7377712 33012377

[20] Comas-Herrera A, Zalakain J, Litwin C, Hsu A, Lane N, Fernández J, Mortality associated with COVID-19 outbreaks in care homes: early international evidence. 2020 Available at: https://ltcCOVID.org/wp-content/uploads/2020/05/Mortality-associated-with-COVID-3-May-final-6.pdf (last accessed 6 July 2020).

[21] Raisi-Estabragh Z, McCracken C, Bethell MS, Cooper J, Cooper C, Caulfield MJ, et al. Greater risk of severe COVID-19 in black, Asian and minority ethnic populations is not explained by cardiometabolic, socioeconomic or behavioural factors, or by 25(OH)-vitamin D status: study of 1326 cases from the UK Biobank. J Public Health (Oxf). 2020;42:451–60. 32556213 10.1093/pubmed/fdaa095 PMC7449237

[22] Lassale C, Gaye B, Hamer M, Gale CR, Batty GD. Ethnic disparities in hospitalisation for COVID-19 in England: the role of socioeconomic factors, mental health, and inflammatory and pro-inflammatory factors in a community-based cohort study. Brain Behav Immun. 2020;88:44–9. 32497776 10.1016/j.bbi.2020.05.074 PMC7263214

[23] Williamson EJ, Walker AJ, Bhaskaran K, Bacon S, Bates C, Morton CE, et al. Factors associated with COVID-19-related death using OpenSAFELY. Nature 2020;584:430–6. 32640463 10.1038/s41586-020-2521-4 PMC7611074

[24] Statistiska Centralbyrån (SCB) Utrikes födda i Sverige [People born abroad in Sweden]. 2020 Available at: https://www.scb.se/hitta-statistik/sverige-i-siffror/manniskorna-i-sverige/utrikes-fodda/ (last accessed 21 July 2020).

[25] Röstlund L, Gustafsson A. De valdes bort av vården – fast vårdplatser stod tomma [They have been prioritized away – despite available hospital beds]. Dagens Nyheter; 6 June 2020.

[26] Radio S. Hård kritik mot äldreboenden: snarare dödshjälp än vård 22 maj 2020 [Harsh criticism of nursing homes: “it is euthanasia rather than care”]. 2020 Available at: https://sverigesradio.se/artikel/7479262 (last accessed 22 May 2020).

